# Probability Characteristics of a Crack Hitting Spherical Healing Agent Particles: Application to a Self-Healing Cementitious System

**DOI:** 10.3390/ma15207355

**Published:** 2022-10-20

**Authors:** Shannon Guo, Samir E. Chidiac

**Affiliations:** Department of Civil Engineering, McMaster University, Hamilton, ON L8S 4L8, Canada

**Keywords:** 3D geometric model, statistical model, self-healing cementitious material, spherical capsules, fill ratio, hit probability

## Abstract

A geometric model is developed to statistically study the probability characteristics of crack intersecting self-healing capsules with a structured random distribution in a cement paste mix. To evaluate the probability of a crack intersecting encapsulated particles, the fill ratio of the crack, and the depth of the first-hit capsule, Monte Carlo simulations are performed. The variables are the crack geometry, i.e., width, length, depth, orientation, skewness, and so on; the size and mass fraction of healing capsules; and the agglomeration of capsules. Models based on statistical analyses for hit probability $P_{h}$, crack fill ratio $R_{f-95}$ at 95% confidence level, and first hit depth $h_{0-95}$ at 95% confidence level are expressed as functions of capsule size and mass fraction, as well as crack geometry. The model assumptions and results are evaluated using data reported in the literature. The data include results from experimental and theoretical studies.

## 1. Introduction

Cracks are detrimental to the durability and load-bearing capacity of concrete structures. Cracks, which initiate from early age as a result of shrinkage, thermal expansion, and/or accidental loading [1,2], allow the ingress of water and deleterious liquids into the concrete core. The interactions between theses liquids and cement, aggregate, and/or steel reinforcements, being chemical, physical, and/or electrochemical reactions, are the main causes of concrete damage and the service life shortening of concrete structures [3,4].

The intrinsic properties of concrete enable autogenous healing of microcracks up to 150 µm [5,6]; however, the healing effectiveness is limited by the availability of unreacted cement particles and water. As such, the need for a more robust and consistent self-healing system has motivated the development of capsule-based autonomous healing that utilizes healing agents encapsulated in micro-capsules dispersed throughout the concrete [7,8,9]. When concrete cracks, capsules intersected by cracks will rupture and release healing agents into the crack, effectively binding the crack, preventing further crack growth and sealing the crack opening to facilitate recovery of durability and mechanical properties in the damaged region [10,11]. However, capsules dispersed throughout the concrete are equivalent to capsule-shaped voids that have adverse effects on the physical and mechanical properties of concrete, particularly the compressive strength [12,13,14]. As such, there is a need for a design methodology that provides a balance between the adverse effects and benefits of adding capsules with healing agents to concrete.

Widespread implementation of capsule-based self-healing concrete still faces many challenges, such as the cost of capsules and lack of standardized design and testing [9,15]. The design of a self-healing concrete system depends on numerous factors, which include but are not limited to the characteristics of the healing agent, e.g., healing agent material, and healing conditions; the capsule, e.g., size, shape, shell thickness, material, and mass fraction; cementitious properties, e.g., cementing material, mix design, and mechanical properties; and the features of the crack, e.g., length, width, tortuosity, mechanism of formation, and propagation. As these factors are cofounding and somewhat interdependent, they exhibit substantial complexity with regards to their effect on self-healing efficiency. As such, it is challenging to investigate self-healing systematically via experimental study for the purpose of developing tools for designing an efficient healing system.

A number of analytical models have been proposed to estimate concrete healing efficiency by studying the optimal dosage of capsules required to heal randomly generated cracks [16,17,18,19,20]. These studies are limited to simple planar crack shape or non-spherical capsules, and do not account for more complex 2D or 3D cracks, capsule distributions, and/or agglomeration of capsules during mixing and placement. Lin et al. [20] proposed a model, based on geometric probability, to estimate the dosage of randomly distributed tubular capsules. The model accounts for 2D surface level crack patterns, but does not consider crack depth and distribution of capsules along the depth of the crack. Zemskov et al. [19] put forward two analytical models that were derived from geometric probability for predicting the probability of capsules intersecting a crack in 2D space, which accounts for crack depth, capsule radius, and volume fraction of capsules. The results are limited to the intersection between a single planar vertical crack and capsules randomly distributed in the cross section of a cube, producing probability contour plots for hitting probability in a three-layer cube. Zhang and Qian [21] also developed a geometric probability model that captures the number of capsules on a planar crack concrete surface. The influence of capsule size, capsule dosage, and crack irregularity was considered. The model was experimentally validated using large size capsules with diameters of 4 mm and 15 mm. Others have developed numerical simulations to account for the random nature of capsule distribution and cracks’ geometry. Huang and Ye [22] employed Monte Carlo simulations to determine the probability of a crack hitting capsules and used a beam model of 40 mm × 40 mm × 160 mm to evaluate the healing efficiency. The probability of the crack hitting capsules was defined as the probability of capsules centered across an “influence zone” around the crack while assuming a 2D cross section, a single planar crack spanning the entire cross section, and randomly dispersed capsules. The effects of capsule size and dosage on hitting probability and healing efficiency were investigated. However, the effects of crack length and width on healing efficiency were not considered.

Pan and Schlangen [23] performed a 3D numerical simulation to determine the probability characteristics of a crack hitting capsules for self-healing concrete. The capsules were randomly and uniformly placed in a cubic representative volume element (RVE) with edge length *L*. The crack was represented by a vertical V-shaped plane and propagates from the edge of the cubic RVE. The fill ratio was determined using selected values of volume fraction of capsule, crack depth, and diameter of spherical capsules. The simulation results showed that the self-healing efficiency improves when using larger capsules, but with an increase in the coefficient of variation. The study was limited to 1 mm and 5 mm diameter capsules, which are significantly larger than the typical capsule sizes used in experiments and practice [15]. Furthermore, the assumed crack geometry is inconsistent with observed irregular crack patterns, particularly in early age concrete.

Motivated by the above noted findings, a study was undertaken to develop a statistical model for determining the probability of an early age crack in a cement matrix intersecting randomly distributed spherical capsules in 3D space. As such, the model focuses on surface cracks with crack dimensions typical of early age cracking. Computer-generated 3D cross sections of cementitious material with a structured random distribution of spherical capsules and a single random surface crack are used to develop probability distribution functions (PDFs) for three critical parameters: the probability of the crack intersecting a capsule; the maximum healing ratio evaluated as the volume of released healing material per unit volume of crack; and the depth at which a capsule is first intersected. PDFs are then used to investigate the effects of capsule size and dosage on crack sealing for different sizes of cracks. For validation, the model results are compared to both experimental and analytical data reported in the literature.

## 2. Numerical Model

### 2.1. Problem Statement

**Figure 1:**
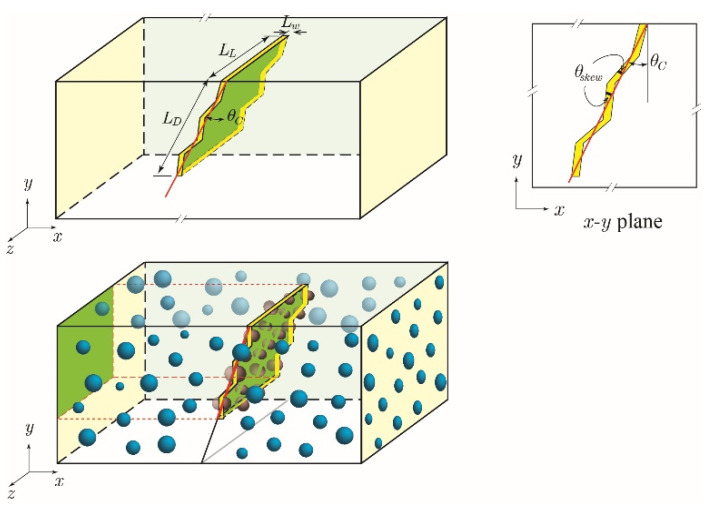
Idealized crack and definition of crack geometry.

The 3D domain, illustrated in Figure 1, consists of cement paste or mortar, a surface crack with a constant width, and mono-sized spherical capsules, with the latter following a structured random distribution. The crack tortuosity is included and characterized by the number of segments (*n_skew_*) and the angles (*θ_skew_*), where *θ_skew_* corresponds to the deviation the propagating crack makes relative to its initial angle *θ_c_* from the *y*-axis. Moreover, the aggregates are not specifically considered. The presence of fine aggregates is assumed to have a minor effect on the capsules’ randomized distribution and the initiation and propagation of surface cracks. As for assessing the effectiveness of the self-healing system, the following measurements are compiled: (1) capsule hit probability, which is the likelihood of a single randomly oriented crack to intersect at least one capsule; (2) depth of first capsule hit, which is a measure of the unhealed crack depth or, in other words, the depth at which a crack will first intersect a capsule and initiate healing at that location; and (3) the fill ratio, which is the ratio between the total volume of the encapsulated healing agent in hit capsules to the total volume of the crack.

### 2.2. Design of Experiment

Circumscribed central composite (CCC) design of experiment (DoE), which is a second-order fractional factorial design, is selected to design the numerical experiments for the purpose of constructing the probability distribution function of healing capability corresponding to capsule hit probability. The DoE accounts for five factors: capsules’ mass fraction *m_f_*, capsule diameter *d*, crack width *L_w_* and depth *L_D_*, and length *L_L_*. Accordingly, 43 combinations of factors are considered, in addition to 16 replicates at the center point to allow for a more uniform estimate of the prediction variance over the entire design space. Table 1 provides the levels selected for each factor. The range of crack dimensions reflects typical crack opening and depth of early age microcracks caused by drying [24] or thermal shrinkage [25]. For each combination, 500 numerical simulations are performed wherein the random variables *δ_i_* (perturbation of capsule position), *n_skew_*, *θ_skew_*, and *θ_c_* are varied within their corresponding range, given in Table 2 and based on a uniformly distributed random distribution. Typical capsule properties, also given in Table 2 [10,12,26], are adopted in this numerical experiment. The measured responses per simulation are as follows: (1) the number of capsules hit by the crack; (2) the depth of the first capsule hit by the crack; and (3) the total volume of capsules hit by the crack.

### 2.3. Geometric Model

**Figure 2:**
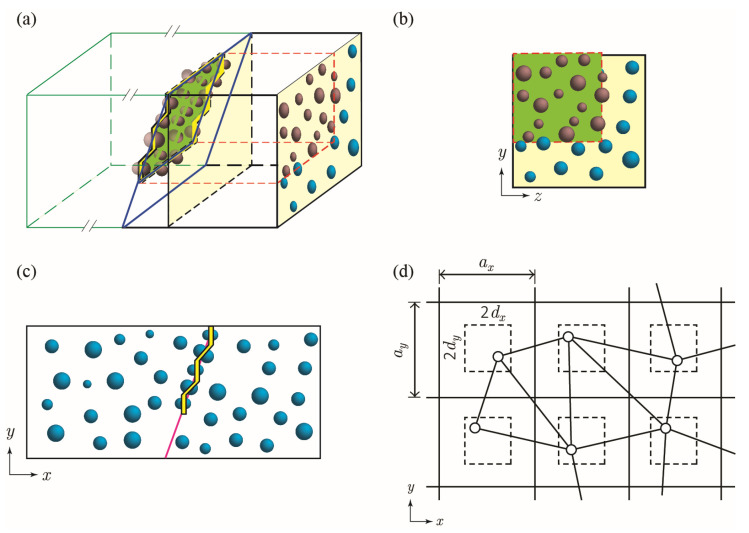
Three-dimensional (3D) visualization of cross section planes with respect to crack placement. (**a**) View of crack plane, (**b**) View of *y*–*z* plane with projection of crack; (**c**) View of *x*–*y* plane; (**d**) Schematic diagram of structured random distribution on *x*–*y* plane.

The 3D domain of a cracked section is modelled using two intersecting 2D cross sections of cement paste containing a structured random distribution of capsules owing to a single surface crack, as shown in Figure 2. These cross sections are randomly generated based on factor levels and variable values given in Table 1 and Table 2 using MATLAB [29].

#### 2.3.1. Capsule Distribution

Capsule distribution is assumed to be statistically equivalent in all directions. The *x*–*y* plane represents capsule distribution perpendicular to the crack face, and the *y*–*z* plane represents capsule distribution on the slanted crack face projected onto the *y*–*z* plane. Randomness of the capsule location is achieved by generating a uniform alternating distribution of capsules and applying a random perturbation in the horizontal (*d_x_* or *d_z_*) and vertical (*d_y_*) directions, as illustrated in Figure 2d. Perturbation of each capsule is assumed to be independent of other capsules, with the position being determined as follows:

(1) $$P_{i,random}=P_{i,regular}+\delta_{i}\;\mathrm{with}\;i=x,\;y,\;z$$

in which $P_{i,regular}$ and $P_{i,random}$ are the capsule coordinates with regular and random distributions, respectively, and $\delta_{i}$ is the capsule perturbation in the in the *x*-, *y*-, and *z*-axis, respectively. To avoid overlap of capsules, $\delta_{i}$ is selected as $\delta_{i}=\delta_{0i}\;\mathrm{rand}\left[-1,1\right]$, with $\delta_{0i}$ being the allowable maximum deviation in the *i*th axis direction and $\mathrm{rand}\left[-1,1\right]$ a random number generated in the range of $\left[-1,1\right]$.

#### 2.3.2. Crack Generation

A surface crack initiating from the top edge of the cement section is generated at a random location. For a conservative purely geometric approach, crack formation is assumed to be independent of other cracks and the inclusion of the presence of capsules. The crack is generated at a random angle *θ_c_* between 0° (parallel to *y*-axis) and 45° from the vertical, with an angle *θ_skew_* between zigzag crack segments and the overall crack propagation direction, as illustrated in Figure 1. For ease of computation, crack width is assumed to be constant, i.e., not tapered along the depth in the *x*–*y* plane direction and along the length in the *z*-direction.

#### 2.3.3. Agglomeration

Although agglomeration and sedimentation of capsules do not have a significant role at low concentrations, capsules’ clustering and crowding are expected to take place at high packing fractions [14]. An agglomeration curve as a function of capsule dosage is introduced to account for the effects of capsule agglomeration. This function is assumed to have the general form:

(2) $$f_{agg}=Ae^{B\left(m_{f}-C\right)}$$

in which *f_agg_* is the total number fraction of capsules to be agglomerated and *m_f_* is the original mass fraction of capsules with respect to cement. Constants *A*, *B*, and *C* are assumed to be *A* = 1, *B* = 20, and *C* = 0.1 to produce an agglomeration curve that reflects capsule clustering trend observed in experimental studies [14]. Total agglomeration is limited to a maximum of 80%, i.e., at least 20% mass fraction of capsules are not in agglomerated clusters.

The size distribution of agglomerates is calculated based on the general function derived for agglomeration of particles in turbulence [30]:

(3) $$f_{i}=\frac{n_{i}}{n_{0}}=\beta \exp(-\frac{i}{\kappa })$$

in which $\beta =2\cosh(\kappa^{-1})-2$, *i* is the agglomerate size (number of capsules in one agglomerate), *n_i_* is the number of agglomerates of size *i*, and *n*_0_ is the original number of non-agglomerated capsules. *κ* is a constant determined by rearranging Equation (3) via

(4) $$\kappa =ln{\left(\frac{-1-\sqrt{f_{1}}}{f_{1}-1}\right)}^{-1}$$

in which $f_{1}=1-\sum f_{agg}$.

### 2.4. Statistical Model

Monte-Carlo simulation was carried out to compute the hit probability $P_{h}$, the probability distribution of hit depth $h_{0}$, and crack fill ratio $R_{f}$ for each combination of the five selected variables. The probability density function (PDF) and the cumulative distribution function (CDF) for the distributions of $h_{0}$ and $R_{f}$ were then determined to estimate their threshold values with 95% confidence $h_{0-95}$ and $R_{f-95}$, respectively. $h_{0-95}$ is defined as the hit depth at which there is 95% probability the first hit depth will be within this value. $R_{f-95}$ is defined as the fill ratio at which there is 95% probability the expected fill ratio will not be less than this value.

#### 2.4.1. Capsule Hit Probability $P_{h}$

Capsule hit probability $P_{h}$ is defined as the probability of a single crack intersecting at least one capsule on the *x*–*y* plane. The perpendicular cut is assumed to be statistically representative of capsule distribution and hit probability along the depth of the crack. Accordingly, the probability of successful capsule intersection on the *x*–*y* plane yields a conservative estimate of the overall hit probability. For each combination of $m_{f},d,L_{W},L_{D},L_{L}$, $P_{h}\left(m_{f},d,L_{W},L_{D},L_{L}\right)$ is determined as

(5) $$P_{h}=N_{hit}/N_{total}$$

in which $N_{total}$ is the total number of simulations and $N_{hit}$ is the number of simulations for which at least one capsule is hit by the crack. $N_{total}=500$ is adopted for this experiment from a series of trial simulations.

#### 2.4.2. First Hit Depth $h_{0}$

**Figure 3:**
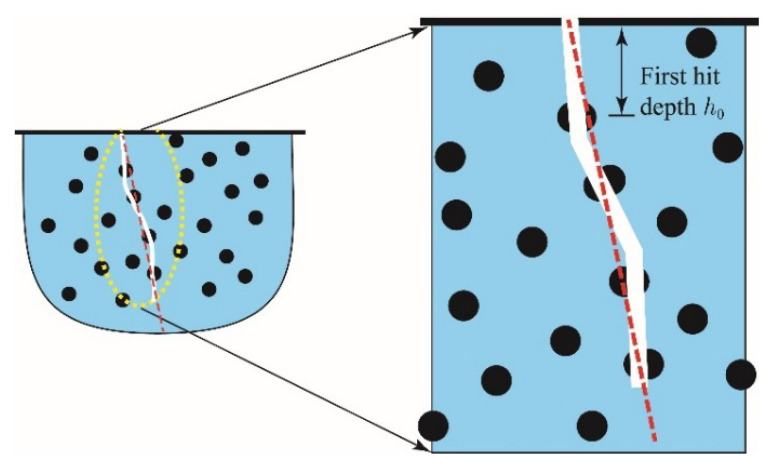
Depth of first hit measured from the top surface.

**Figure 4:**
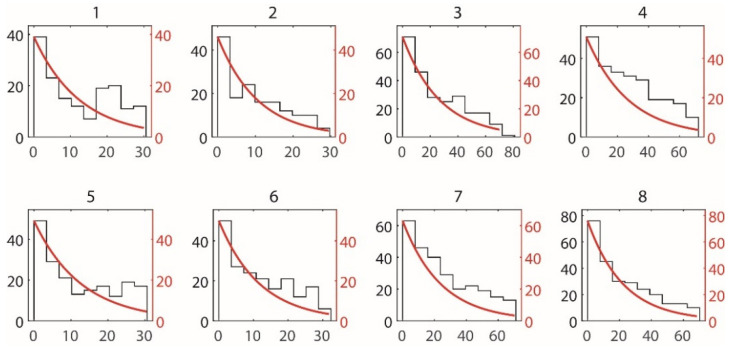
Frequency distribution of hit depth for different combinations of variable levels.

The first hit depth $h_{0}$, shown in Figure 3, is the distance from the top surface to the position of the crack intersecting the first capsule. $h_{0}$ is valid only in the case when the crack successfully intersects with a capsule, thus only trials with successful capsule intersection (i.e., where crack fill volume is greater than zero) were used in the analysis of hit depth. To ensure consistent statistical power, additional trials were run where necessary to ensure each combination has a minimum of 100 trials with successful intersection and $h_{0}$ data. Figure 4 shows typical frequency distributions of $h_{0}$. An Anderson–Darling (AD) test [31] for distribution type reveals that the distribution of $h_{0}$ for each combination generally follows a Weibull distribution (α = 0.05) with the PDF $f_{X}\left(x|\lambda \right)$ and CDF function $F\left(x\right)$ being

(6) $$f_{X}\left(x|\lambda \right)=\left\{\begin{array}{cc} \frac{k}{\lambda }{\left(\frac{x}{\lambda }\right)}^{k-1}e^{-{\left(x/\lambda \right)}^{k}} & \;\mathrm{for}\;x\geq 0\\ 0 & \;\mathrm{for}\;x<0 \end{array}\right.$$

with $E\left[X\right]=\lambda^{-1}$, λ>0, and

(7) $$P\left(X\geq x^{*}\right)=\int_{x^{*}}^{\infty }\frac{k}{\lambda }{\left(\frac{x}{\lambda }\right)}^{k-1}e^{-{\left(x/\lambda \right)}^{k}}dx=e^{-{\left(x/\lambda \right)}^{k}}$$

The threshold value $x^{*}$ corresponding to $P\left(X\geq x^{*}\right)$ yields the value of $h_{0-95}$ with 95% confidence with $P(X>x^{*})=95\%$. Similar to hit probability, $h_{0-95}$ is a function of $m_{f},d,L_{W},L_{D},L_{L}$.

#### 2.4.3. Crack Fill Ratio $R_{f}$

**Figure 5:**
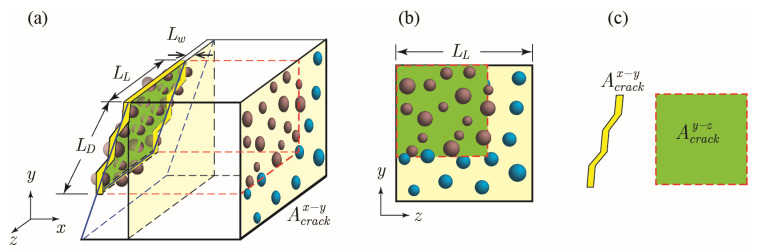
Characteristics of a crack related to the calculation of crack fill ratio. (**a**) View of crack plane; (**b**) View of *y*–*z* plane with projection of crack; (**c**) Definition of $A_{crack}^{x-y}$ and $A_{crack}^{y-z}$.

The crack fill ratio $R_{f}$ is the ratio of the healing agent released into the crack relative to the total crack volume. As illustrated in Figure 5a,b, the total volume of a crack can be determined as

(8) $$V_{crack}=A_{crack}L_{w}=A_{crack}^{x-y}L_{L}=L_{L}L_{D}L_{w}/\cos\theta_{skew}$$

For uniform distribution of capsules, the number of capsules intersecting a crack is proportional to $A_{crack}$ and independent of crack orientation. Given the projection of $A_{crack}$ on the *y*–*z* plane $A_{crack}^{y-z}$ and the number of capsules $n_{proj}^{y-z}$ in the range of $A_{crack}^{y-z}$ on the *y*–*z* plane, the crack fill ratio is approximated by

(9) $$R_{f}=\frac{V_{0}n_{proj}^{y-z}}{\;A_{crack}}=\frac{V_{0}n_{proj}^{y-z}}{A_{crack}^{x-y}L_{L}}=\frac{V_{0}n_{proj}^{y-z}}{A_{crack}^{x-y}}\frac{L_{D}cos\theta_{skew}}{A_{crack}^{y-z}}$$

in which *v*_0_ is the total volume of healing agent released into the crack.

**Figure 6:**
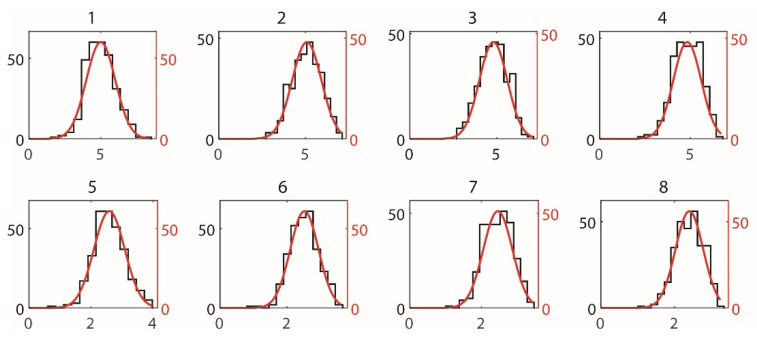
Frequency distribution (*x*-axis, number of occurrence) of fill ratio (*y*-axis, %) for different combinations of variable levels.

Figure 6 presents typical frequency distributions of $R_{f}$. It is found that the distribution of $R_{f}$ generally follows normal distribution (α = 0.05), with the PDF and CDF being

(10) $$f_{X}(x|\mu ,\sigma^{2})=\frac{1}{\sigma \sqrt{2\pi }}e^{-\frac{1}{2}{\left(\frac{x-\mu }{\sigma }\right)}^{2}}$$

and

(11) $$P\left(X\geq x^{*}\right)=F\left(x^{*}\right)=\frac{1}{\sigma \sqrt{2\pi }}\int_{x^{*}}^{\infty }e^{-\frac{{\left(x-\mu \right)}^{2}}{2\sigma^{2}}}dx$$

Subsequent to determining the mean *µ* and the standard deviation *σ*, the threshold value $x^{*}$ corresponding to $P(X>x^{*})$ yields the value of $R_{f-95}=R_{f-95}\left(m_{f},d,L_{W},L_{D},L_{L}\right)$ with 95% confidence when $P(X>x^{*})=95\%$.

### 2.5. Regression Analysis

Regression analyses for the Monte-Carlo simulation results were carried out to determine mathematical expressions for $P_{h}$, $h_{0-95}$, and $R_{f-95}$ as functions of the five independent variables. A stepwise regression analysis is performed as follows:

1. Perform a linear regression analysis

(12) $$Y^{\left(k\right)}=a_{0}^{\left(k\right)}+\sum_{i=1}^{5}a_{i}^{\left(k\right)}x_{i}^{\left(k\right)}$$

in which $x_{i}^{\left(k\right)}$ stands for the *i*th variable for $Y^{\left(k\right)}$; $a_{0}^{\left(k\right)}$ and $a_{i}^{\left(k\right)}$ are regression coefficients; and *k* = *h*, *d*, and *f*, respectively. The results are then used as a reference to select the best regression model.

2. Perform a complete quadratic polynomial regression analysis

(13) $$Y^{\left(k\right)}=a_{0}^{\left(k\right)}+\sum_{i=1}^{5}a_{i}^{\left(k\right)}x_{i}^{\left(k\right)}+\sum_{\begin{array}{c} i=1\\ j\geq i \end{array}}^{5}b_{ij}^{\left(k\right)}x_{i}^{\left(k\right)}x_{j}^{\left(k\right)}$$

The second-order terms $b_{ij}^{\left(k\right)}x_{i}^{\left(k\right)}x_{j}^{\left(k\right)}$ with j≠i reflect the interaction between different variables. Interaction implies that the relationship between $Y^{\left(k\right)}$ and $x_{i}^{\left(k\right)}$ changes with a third variable $x_{j}^{\left(k\right)}$. A *t*-statistic analysis is conducted to determine the *p*-value for each coefficient, where a *p*-value greater than the critical value suggests that the corresponding term is statistically insignificant for the regression.

3. Stepwise, eliminate the terms in Equation (13) with *p* > 0.05, starting off with terms having higher values of *p*, e.g., when *p* > 0.1, to optimize the regression relationship.

4. Compare the values of $Y^{\left(k\right)}$ obtained from Monte-Carlo simulations with regression for various regression relations and check the coefficient of multiple determination *R^2^* value as well as the relative error distribution.

5. Select the most representative regression expression by examining error distribution and *R*^2^ of the estimators.

#### 2.5.1. Hit Probability $P_{h}$

Regression models suggest that crack length ($x_{5}$) has a negligible influence on hit probability. Coefficient values show that hit probability generally increases as the mass fraction, crack width, and crack depth increase. An increase in the capsule size with other quantities, i.e., mass fraction and remaining constant, tends to result in a lower hit probability. The t-statistic and *p*-values show that most variables have a negligible interaction; however, mass fraction and crack depth show a weak interaction represented by the term $x_{1}x_{4}$. After eliminating all terms with *p* > 0.1 and considering *x*_1_*x*_4_ as a potential candidate, a final non-linear expression is selected based on simplicity and distribution of errors:

(14) $$\mathrm{Model}\;-\;P_{h}:\;Y=\left(a_{1}x_{1}+a_{2}x_{2}+a_{3}x_{3}+a_{4}x_{4}+a_{0}\right)+b_{11}x_{1}^{2}+b_{44}x_{4}^{2}$$

The corresponding *R*^2^ values and the maximum *p*-value of coefficients are 0.94 and 10^−5^, respectively, for model-P_h_. Table 3 summarizes the regression results of the model.

**Figure 7:**
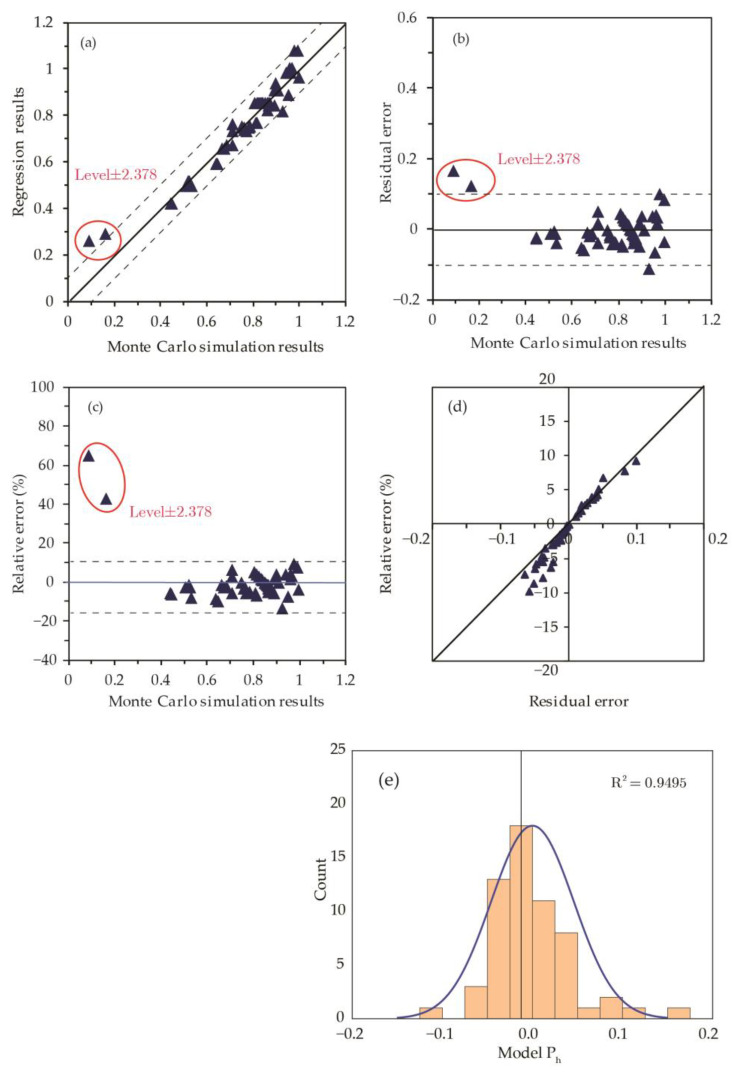
Comparison of regression models for P_h_. (**a**) Regression and Monte-Carlo simulation results; (**b**) residual error; (**c**) relative error; (**d**) relative error against residual error; and (**e**) relative error distribution for P_h_.

Figure 7 presents model-P_h_ in terms of fit with numerical simulation results, residual error, and relative error distributions. By examining the results, the following conclusions are deduced: the regression model predictions have a maximum residual error less than ±0.100 except for two data points that correspond to extreme variables value (i.e., level ±2.378), and the model *R*^2^ value is 0.95.

**Figure 8:**
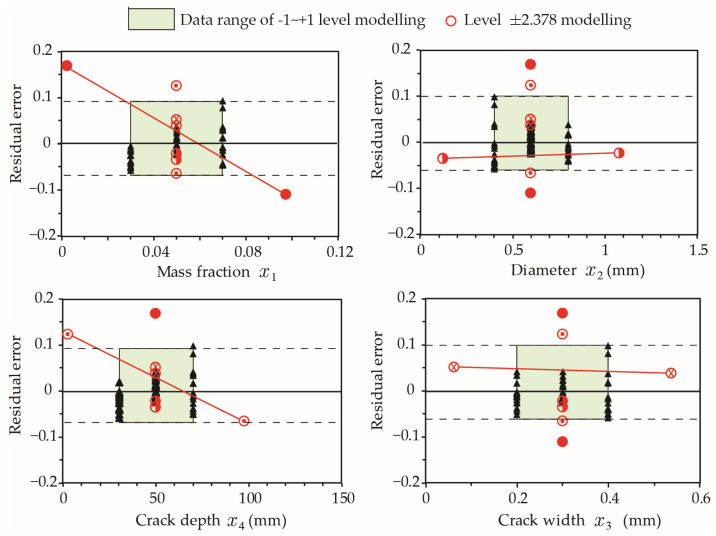
Influence of individual factors on residual errors of model-P_h_. The results of extreme cases at Level ±2.378 are connected by a red line.

Given that the errors at extreme values corresponding to levels ±2.378 are greater than 10%, it is necessary to examine model-P_h_ results when values of *x_i_* (*i* = 1 to 4) are out of the typical range specified by levels [−1, +1] in Table 1. Figure 8 presents the model relative errors at extreme values. The results reveal a higher residual error for mass fraction ($x_{1}$) and crack depth ($x_{4}$) when their values are outside [−1, +1] levels, while variations in capsule diameter ($x_{2}$) and crack width ($x_{3}$) result in a residual error typical of the model estimate. Accordingly, model-P_h_ should be limited to $0.02<x_{1}<0.08$ and $20\;\mathrm{mm}<x_{4}<100\;\mathrm{mm}$ for mass fraction and early age crack depths, respectively.

#### 2.5.2. First Hit Depth $h_{0-95}$

Following the same procedure for the determination of hit probability, regression analyses are carried out to determine the best estimate for $h_{0-95}$ as a function of $\left(m_{f},d,L_{W},L_{D},L_{L}\right)$, or $Y_{f95}=Y_{f}\left(x_{1},x_{2},x_{3},x_{4},x_{5}\right)$. The model results suggest that crack length ($x_{5}$) has a negligible influence on $h_{0-95}$. The sign of coefficients indicates that $h_{0-95}$ tends to decrease when increasing the mass fraction of capsules for a wider crack. However, at a fixed mass fraction, the use of larger diameter capsules yields larger $h_{0-95}$, suggesting that a lower fraction of capsules will increase the crack depth required until a capsule is encountered. The selected model takes the following form:

(15) $${\mathrm{Model}\;-\;H}_{0}:\;Y=a_{4}x_{4}+b_{12}x_{1}x_{2}+b_{14}x_{1}x_{4}+b_{22}x_{2}^{2}+b_{24}x_{2}x_{4}+b_{34}x_{3}x_{4}+b_{44}x_{4}^{2}$$

**Figure 9:**
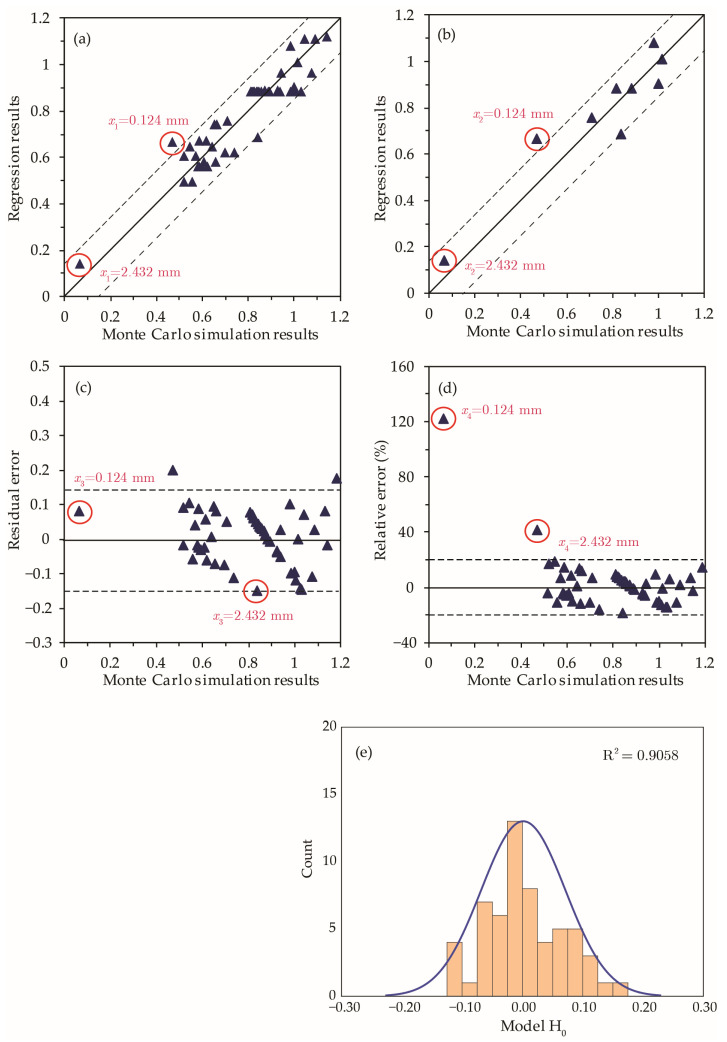
Comparison of different regression models for: (**a**) regression and Monte-Carlo simulation results; (**b**) regression and Monte-Carlo simulation results for level ±2.378 simulations; (**c**) residual error; (**d**) relative error; and (**e**) residual error distribution.

The corresponding *R*^2^ values and the maximum *p*-value of coefficients are 0.91 and 0.01, respectively, for model-H_0_. The majority of the residual errors are less than 0.01 for points within levels [−1, +1], as shown in Figure 9a,c. A larger residual error and relative error are observed for extreme values of *x*_2_ and *x*_4_ at level ±2.378, notably for small diameters ($x_{2}=0.124\;\mathrm{mm}$) and short cracks ($x_{4}=2.432\;\mathrm{mm}$), as illustrated in Figure 9b–d. Table 4 summarizes the regression results of model-H_0_.

**Figure 10:**
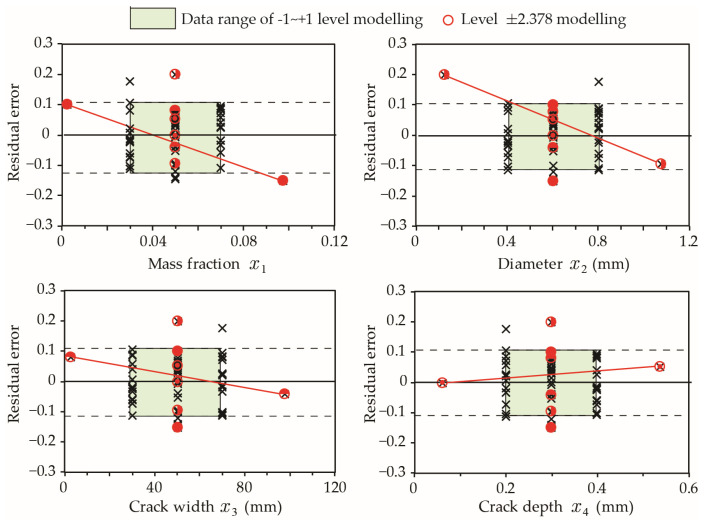
Influence of individual factors on residual errors of model-H_0_. The results of extreme cases at Level ±2.378 are connected by a red line.

Similar to model-H_0_ for hit probability presented in Equation (14), model-H_0_ for hit depth $h_{0-95}$ has a higher residual error $\Delta h_{0-95}$ when estimating the results at extreme input values (level ±2.378). The sensitivity of $\Delta h_{0-95}$ to variables $x_{1}$ to $x_{4}$ is illustrated in Figure 10. It is evident that $\Delta h_{0-95}$ for higher mass fractions or small size capsules significantly exceeds the typical $\Delta h_{0-95}$ range of level [−1, +1] points. Accordingly, it is not recommended to use model-H_0_ represented by Equation (15) when mass fraction $x_{1}>8\%$ and capsule diameter $x_{2}>0.3\;\mathrm{mm}$.

#### 2.5.3. Crack Fill Ratio $R_{f-95}$

Likewise, regression analysis was carried out to determine best estimate of $R_{f-95}$ as a function of $Y_{f95}=Y_{f}\left(x_{1},x_{2},x_{3},x_{4},x_{5}\right)$. The initial model results suggest that $R_{f-95}$ can be considered independent of crack depth $x_{4}$ and crack length $x_{5}$. In general, the value of $R_{f-95}$ tends to increase for high mass fraction and large size capsules, while decreasing for a wide crack. The depth and length of the crack have a negligible effect on $R_{f-95}$, because the number of capsules intersecting a crack is generally proportional to the crack face area, which is determined by the crack depth and length. By iteratively eliminating terms with high *p*-values and examining the relative error of potential models, an optimized quadratic expression with *R*^2^ = 0.93 is obtained and given by the following:

(16) $$\mathrm{Model}\;-\;R_{f}:\;Y=a_{0}+a_{3}x_{3}+b_{12}x_{1}x_{2}+b_{13}x_{1}x_{3}+b_{22}x_{2}^{2}+b_{23}x_{2}x_{3}+b_{33}x_{3}^{2}$$

**Figure 11:**
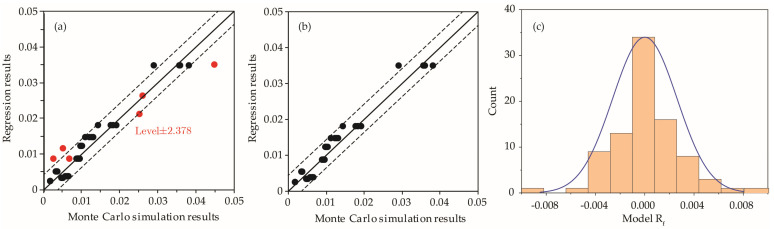
(**a**) Regression and Monte-Carlo simulation results with all data points displayed; (**b**) regression and Monte-Carlo simulation results with extreme mass fraction and diameter removed from the plot; and (**c**) residual error distribution for model-R_f_.

The summary of regression results for model-R_f_ is presented in Table 5. Figure 11 presents the distribution of errors associated with the model-R_f_ regression model. It should be noted that the distribution of errors in Figure 11 does not show two extreme points at level ±2.378 for high mass fractions and small size capsules.

**Figure 12:**
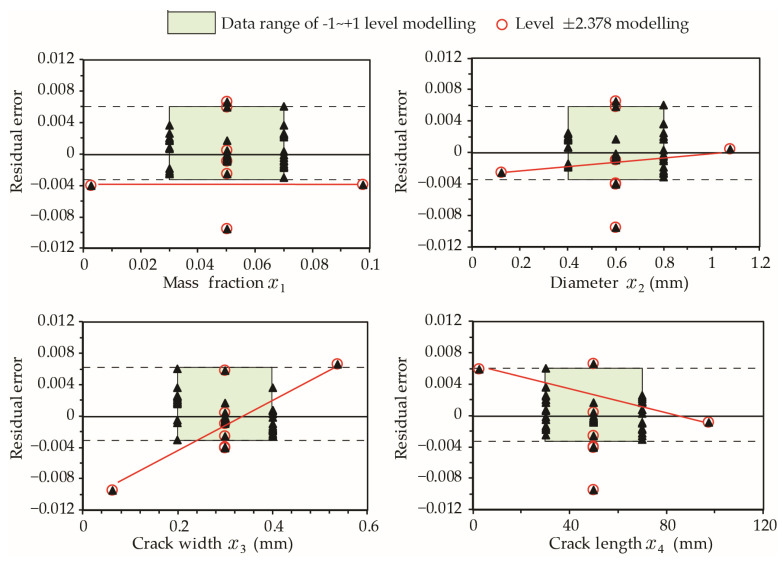
Influence of individual factors on residual errors for $R_{f-95}$. The results of extreme cases at Level ±2.378 are connected by a red line.

**Figure 13:**
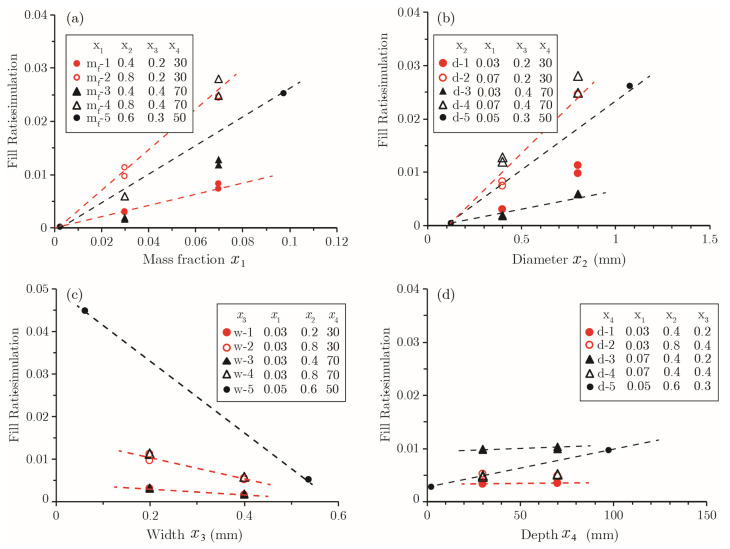
Variation of $R_{f-95}$ with different variables under various combinations.

Figure 12 summarizes the $R_{f-95}$ sensitivity analysis results for model-R_f_. The residual error $\Delta R_{f-95}$ is not as sensitive to larger variations in other variables; however, caution should be used in situations of smaller crack depths. $\Delta R_{f-95}$ for very large or very narrow crack widths significantly exceeds the typical $\Delta R_{f-95}$ range of level [−1, +1] points. As such, it is not recommended to use model-R_f_ for very short and long cracks lying outside the noted size range of ($0.1<x_{4}<0.5$). When adjusting a single variable while holding other variables constant, the value of $R_{f-95}$ tends to increase with the mass fraction and diameter of capsules (Figure 13a,b). As one may expect, the healing effectiveness decreases as the crack becomes wider (Figure 13c). The influence of crack depth on $R_{f-95}$ can be considered negligible, as shown in Figure 13d.

Analyzing the coefficient values of the regression model, the following relationships regarding the coupled effect of different variables can be drawn:

1. To achieve the desired $R_{f-95}$ with a given capsule size $x_{2}$, coefficient values for $x_{1}$ and $x_{3}$ show that the mass fraction of capsules must be increased to heal wider cracks. This is in agreement with the experimental observations [32].
2. For a desired $R_{f-95}$ and when targeting cracks with a specific width *x*_1_, coefficient values for $x_{1}$ and $x_{2}$ show that the overall mass fraction can be reduced when using larger capsules. This conclusion concurs with the findings of Lv et al. [16] and Huang and Ye [22].
3. For a desired $R_{f-95}$ and fixed capsule mass fraction *m_f_*, coefficient values for $x_{2}$ and $x_{3}$ show that large size capsules must be used to heal wider cracks.

The above observations are consistent with findings in the literature [6,7,8,10,11,21,22,33].

## 3. Regression Models’ Evaluation

**Figure 14:**
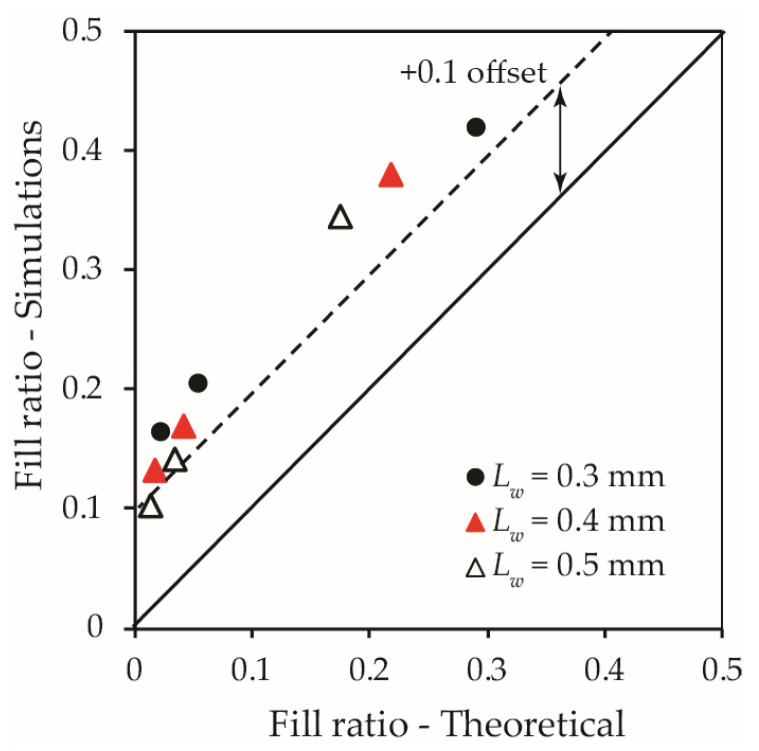
Comparison of the simulation results obtained from numerical modelling to those from theoretical data (Zhang and Qian [21]).

Evaluation of the model assumptions and results is carried out using data reported in the literature. Zhang and Qian [21] carried out series of tests on microbial self-healing concrete, with and without aggregates, using 100 mm × 100 mm × 100 mm cubic specimens. They reported that the number of capsules *k* on a cross-sectional area, which is considered as a planar crack, generally follows a Poisson distribution:

(17) $$f\left(k\right)=\frac{\lambda^{k}}{k!}e^{-\lambda },k=1,2,3\cdots$$

where *λ* is the expected value representing the mean number of capsules intersecting the crack surface and *k* is the variance of the Poisson distribution. The Poisson parameter *λ* is determined by λ=nP, in which *n* represents the number of healing agent particles added into the representative cube and *P* is the probability of a single healing agent particle meeting the crack surface. Alternatively, for mono-sized spherical capsules, *n* can be related to the mass (or volume) fraction of added capsules. With the level of accumulative probability associated with the number of capsules on the crack surface, the value of *k* in Equation (17) can be determined. The fill ratio of a crack can then be evaluated when the crack width is known. Adopting the capsule data in Zhang and Qian [21], Figure 14 compares the fill ratio obtained from the Monte-Carlo simulations in this study with that determined from the theoretical method proposed by Zhang and Qian [21]. The numerical simulation results for fill ratio in this study are consistent in trend with the measured data, which confirms that the modelling method developed in this study is representative and Equation (15) can be used to estimate the fill ratio. It should be noted that the fill ratio obtained from this study is on average 10% higher than theoretical values based on the study by Zhang and Qian [21], which is most likely owing to the tortuosity of the crack considered in this study. Greater crack tortuosity increases the potential intersection region in the vicinity of the crack, resulting in a greater number of capsules being intersected by a single crack. This is in agreement with the observation of Zhang and Qian [21] that an undulating crack shape tends to intersect more capsules than a planar crack.

**Figure 15:**
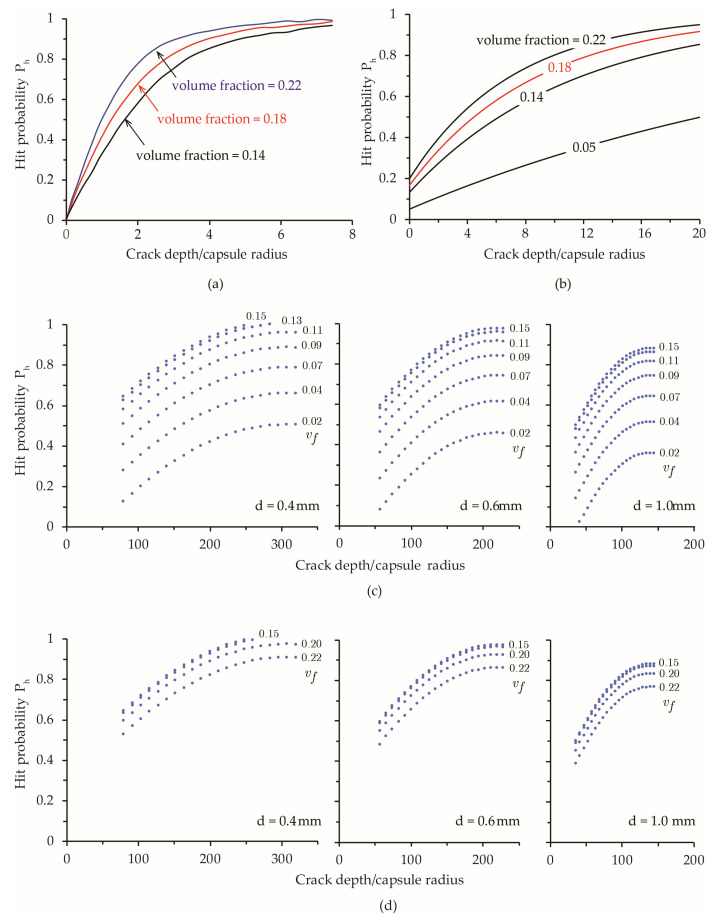
Simulations results by (**a**) Zemskov et al. [19] and (**b**) Lv and Chen [17]; (**c**,**d**) Variation of hit probability predicted by regression model with normalized crack depth, for different capsule volume fractions $\upsilon_{f}=0.02-0.15$ and $\upsilon_{f}=0.15-0.22,$ respectively.

Zemskov et al. [19] investigated the probability of hitting any capsule by a planar crack in self-healing materials. Two mathematical models were developed for 2D layered random placement and fully random placement of capsules, respectively. The models were validated with Monte-Carlo tests for selected conditions, namely, 27 capsules with diameter varying from 2 mm to 4 mm were placed in a cubic specimen with a side length of approximately 10 mm. The corresponding capsules volume fraction $\upsilon_{f}$, which is defined as the total capsule volume/mix volume, ranges between 0.065 and 0.382. Another study by Lv and Chen [17] on the dosage of capsules embedded in self-healing materials examines the influence of crack depth and volume fraction of capsules on hit probability for planar cracks perpendicular to the concrete surface. For 2D cases, the probability of a crack to intersect at least one spherical capsule is given by $P_{h}=1-exp\left[-v_{f}\left(1+\frac{2L_{D}}{\pi R}\right)\right]$, in which $\upsilon_{f}$ and *R* are the volume fraction of capsules and radius of capsules, respectively. Figure 15a,b, reproduced from Zemskov et al. [19] and Lv and Chen [17], respectively, present the probability of hitting any capsule by a planar crack at a different volume fraction $\upsilon_{f}$ of capsules and normalized crack depth. For a given $\upsilon_{f}$, the value of $P_{h}$ increases quickly with crack depth for short cracks and gradually approaches a critical value when the crack is sufficiently long. An increase in $\upsilon_{f}$ tends to result in a higher hit probability, as expected.

Figure 15c,d presents the variation in $P_{h}$ obtained in this study under typical crack depth (*L_D_* = 30–70 mm), capsule diameter (d=0.4−1.0 mm), and mass fraction of capsules (*m_f_* = 1–10%); meanwhile, the probability functions developed by Zemskov et al. [19] were for capsules of size d=2.0 mm and crack depths *L_D_* = 0.05–8.5 mm. The results from Lv and Chen [17] correspond d=2.0 mm and *L_D_* = 0.05–8.5 mm. Given the difference in the range of variables and the assumptions about the cracks, it is not reasonable to compare the results directly. As such, a qualitative/semi-quantitative evaluation is carried out to examine the general trends as well as the similarities and differences in the results.

For a given diameter and volume fraction of capsules, the variation in $P_{h}$ with crack depth is the same as that depicted in Figure 15a,b. The critical $P_{h}$ values for long cracks depend on the volume fraction, which is consistent with the results in Lv and Chen [17], as presented in Figure 15b. When *v_f_* < 5% (or *m_f_* < 7%), such as in Figure 15c, $P_{h}$ increases as *v_f_* increases. However, for the case of *v_f_* > 15% in Figure 15d, $P_{h}$ tends to decrease slightly as *v_f_* increases, likely owing to the effects of capsule agglomeration, which become more pronounced at a high mass fraction according to Equation (2).

**Figure 16:**
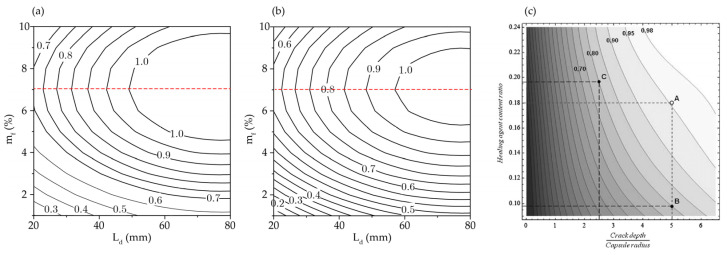
Contour plot of hitting probability $P_{h}$ as a function of *L_D_* and *m_f_* for (**a**) $d=0.2\;\mathrm{mm},L_{w}=0.3\;\mathrm{mm}$ and (**b**) $d=0.6\;\mathrm{mm},L_{w}=0.3\;\mathrm{mm}$; (**c**) Hitting probability as a function of normalized crack depth and volume fraction of capsules, for a cube of side length equivalent to 1cm containing 27 capsules, with V_capsule_/V_cube_ varying from 0.1–0.3 [19].

Figure 16 shows the contour plots of hit probability $P_{h}$ as a function of crack depth and mass fraction. Only numerical simulation results within the typical range of values $L_{w}=0.3\;\mathrm{mm}$, d=0.2 mm, and d=0.6 mm are presented in Figure 16a,b to compare qualitatively with Figure 16c, which is reproduced from Zemskov et al. [19]. When *m_f_* < 7%, the $P_{h}=F\left(L_{D},m_{f}\right)$ contours in Figure 16a,b have the same trend of variation as that in Figure 16c; in particular, the value of $P_{h}$ increases with *L_D_* and *m_f_*. Figure 16a,b show that, at any mass fraction in the range 5% < *m_f_* < 7%, a long crack will hit at least one capsule. For lower mass fractions of capsules, the maximum value of $P_{h}$ may only be reached at certain capsule diameters.

Figure 16a,b reveal the effects of capsule agglomeration when *m_f_* > 7%, which show a deviation from the results of Zemskov et al. [19]. Agglomeration causes capsule clustering and reduces the number density of capsules in the mix, which in turn reduces the probability of a crack hitting a capsule. The agglomeration effect becomes more pronounced with a continual increase in *m_f_*. This trend of $P_{h}$ variation is clearly demonstrated in Figure 16a,b.

**Figure 17:**
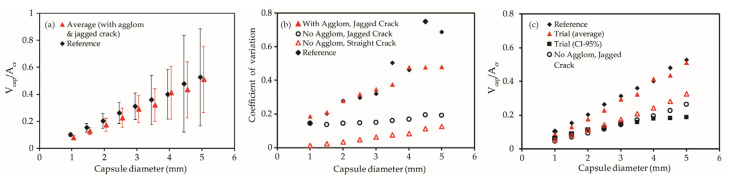
Effect of capsule agglomeration and crack geometry on the variation in fill ratio. (**a**) Comparison of variances between numerical study and simulation; (**b**) Uncertainty in data with different crack geometry; (**c**) Effect of crack geometry on average fill ratio.

A numerical study by Pan and Schlangen [23] investigated the hit probability from a geometric perspective for a cubic mortar RVE containing randomly placed spherical capsules and a vertical V-shaped crack perpendicular to the edge of the RVE. Figure 17 shows the results as reproduced from Pan and Schlangen [23] along with the simulation results obtained using equivalent capsule properties. For a higher number of capsules, the standard deviation of the expected fill ratio increases, thus causing a challenge in selecting a suitable dosage with high certainty. While healing efficiency increased on average with a higher dosage, a noticeably higher uncertainty is also observed at a higher dosage, especially for capsules with diameters of 4.5 mm or greater, as shown in Figure 17a. This can be attributed to the decrease in overall number density of capsules to maintain the same volume ratio, thus increasing variability in the capsule distribution.

Figure 17b,c plot the average fill ratio simulated with the influence of random crack skewness and capsule agglomeration, which share similar values and trends to the results presented by Pan and Schlangen [23]. As expected, the introduction of crack skewness and capsule agglomeration increases the standard deviation of the expected fill ratio. Meanwhile, the average value at 95% confidence has similar values to trials with lower variability owing to agglomeration and crack skewness. The use of the developed models provides a more reliable and conservative design with 95% confidence interval predictions while also considering the effect of capsule agglomeration and crack geometry.

## 4. Conclusions

The efficacy of a self-healing system depends on the probability of hitting a capsule, the filling capacity of the intersected capsules, and the depth at which a capsule is first intersected. These variables are found to be affected by crack geometry and tortuosity, healing capsules’ size, and mass fraction, as well as capsules’ agglomeration. Specifically, the following conclusions are derived from this study:

1. The proposed framework has captured the observations previously reported in the literature, including the effect of capsule size and dosage and crack opening on hit probability, filling ratio, and hit depth.
2. The 95% confidence level adopted in this study is recommended for the design of a self-healing system as it reduces the uncertainties in the design and significantly increases the efficacy of a healing system.
3. Agglomeration with an increasing dosage of capsules reduces hit probability while increasing the crack fill volume. Further addition of capsules past the noted threshold of 7% mass fraction yields adverse effects on hit probability. This shows that agglomeration effects are an important factor that must be considered.
4. Crack tortuosity increases the potential intersection region and results in a higher number of capsules intersected.
5. Irregular cracks have a larger crack volume compared with a straight crack of the same depth, resulting in an overall increase in fill ratio.
6. Higher crack tortuosity slightly increases the uncertainty in the expected fill ratio.

The deduced findings are limited to the variables and results presented in this study. Although this study provides a significant contribution for designing an efficient self-healing cementitious system, it still needs to account for the interaction between the capsule and the cement paste when a crack hits the capsule. In this study, it is assumed that the capsules will rupture when they intersect a crack.

## Figures and Tables

**Table 1:** Variables and levels considered for full five-factor CCC factorial design.

	Level				
Variable	−2.378	−1	0	1	2.378
Capsule Properties					
Mass fraction, *m_f_* (%)	0.243%	3%	5%	7%	9.757%
Diameter, *d* (mm)	0.024	0.3	0.5	0.7	0.976
Crack Properties					
Crack width, *L_W_* (mm)	0.062	0.2	0.3	0.4	0.538
Crack depth, *L_D_* (mm)	2.432	30	50	70	97.568
Crack length, *L_L_* (mm)	6.216	20	30	40	53.784

**Table 2:** Constants and variables considered in the DoE.

Variable	Value
Material Properties	
Cement density	*ρ_cement_* = 3150 kg/m^3^
Water-to-cement ratio	0.5
Capsule core material density (DCPD)	*ρ_core_* = 980 kg/m^3^ [27]
Shell material density (urea-formaldehyde)	*ρ_shell_* = 1170 kg/m^3^ [28]
Shell thickness	*t_shell_* = 1 µm
Domain Properties	
Width of sample area	*L_x_* = 150 mm
Depth of sample area	*L_y_* = 100 mm
Length of sample area	*L_z_* = 150 mm
Perturbation of capsule position	$\delta_{i}=\left[-d_{i}/2,d_{i}/2\right],i=x,y$
Crack Properties	
Angle from vertical (*y*-axis)	*θ_c_* range = [−π/4, +π/4]
Skewness (angle of zigzag segments)	*θ_skew_* range = [0, π/4]
Number of segments of zigzag	*n_skew_* range = [0, 10]

**Table 3:** Summary of regression coefficients for P_h_.

Variables	Coefficient	Value	Standard Error	*t*-Ratio	*p*-Value
-	a_0_	−0.3632	0.0626	−5.8006	0
$100x_{1}$	a_1_	0.1966	0.0155	12.6209	0
$x_{2}$	a_2_	−0.2362	0.0368	−6.4090	0
$x_{3}$	a_3_	0.3714	0.0737	5.0382	10^−5^
$x_{4}$	a_4_	0.0179	0.0015	11.4808	0
$x_{1}^{2}$	b_11_	−0.0138	0.0015	−9.1103	0
$x_{4}^{2}$	b_44_	−0.0001	0.0000	−7.3440	0

**Table 4:** Summary of the regression coefficients for $h_{0-95}$.

Variables	Coefficient	Value	Standard Error	*t*-Ratio	*p*-Value
$x_{4}$	a_4_	0.0274	0.0014	18.8052	0
$x_{1}x_{2}$	b_12_	0.0844	0.0208	4.0569	0.00017
$x_{1}x_{4}$	b_14_	−0.0018	0.0002	−7.2618	0
$x_{2}^{2}$	b_22_	−0.4373	0.1475	−2.9636	0.00458
$x_{2}x_{4}$	b_24_	0.0070	0.0026	2.6739	0.01
$x_{3}x_{4}$	b_34_	−0.0106	0.0024	−4.4041	0.00005
$x_{4}^{2}$	b_44_	−0.0001	0.0001	−3.3545	0.00149

**Table 5:** Summary of regression coefficients for $R_{f-95}$.

Variables	Coefficient	Value	Standard Error	*t*-Ratio	*p*-Value
-	a_0_	0.01661	3.944 × 10^−3^	4.2124	0.00010
*x*_2_	a_2_	0.00829	8.098 × 10^−4^	10.2392	0.00000
*x*_3_	a_3_	−0.00774	1.633 × 10^−3^	−4.7371	0.00002
*x*_1_ *x*_3_	b_13_	−0.00774	1.633 × 10^−3^	−4.7371	0.00002
*x*_2_ *x*_2_	b_22_	0.01566	5.432 × 10^−3^	2.8822	0.00573
*x*_2_ *x*_3_	b_23_	−0.10013	2.000 × 10^−2^	−5.0072	0.00001
*x*_3_ *x*_3_	b_33_	0.26245	3.353 × 10^−2^	7.8267	0.00000

## Data Availability

All data, models, or code that support the findings of this study are available from the corresponding author upon reasonable request.
